# RETRACTION: The Roles of KIFC1 in the Development of Osteosarcoma: Characterization of Potential Therapeutic Targets

**DOI:** 10.1155/cmmm/9852090

**Published:** 2025-05-14

**Authors:** Computational and Mathematical Methods in Medicine

RETRACTION: L.-Y. Liang and G.-S. Li. “The Roles of KIFC1 in the Development of Osteosarcoma: Characterization of Potential Therapeutic Targets,” *Computational and Mathematical Methods in Medicine,* no. 2022 (2022): 1–10. https://doi.org/10.1155/2022/5039134.

The above article [1], published online on 18 April 2022 in Wiley Online Library (http://wileyonlinelibrary.com), has been retracted by John Wiley & Sons Ltd.

The retraction has been agreed following an investigation of the concerns raised by *Hoya camphorifolia* on PubPeer [1], which identified an instance of figure duplication.

Specifically:

-Figure 4b: The Western Blot bands corresponding to KIFC1 and *β*-actin expression in the U-2 OS cells have been duplicated in Figure 5b of [2], where they are labelled as Ki-67 and *β*-actin expression in KIF15-knockout tumors.

As a result of the investigation, the data and conclusions of this article are considered unreliable.

The authors were informed of the decision to retract the article but did not provide a response.
